# Using machine learning models to predict the impact of template mismatches on polymerase chain reaction assay performance

**DOI:** 10.1038/s41598-025-98444-8

**Published:** 2025-05-09

**Authors:** Brittany Knight, Taylor Otwell, Michael P. Coryell, Jennifer Stone, Phillip Davis, Bryan Necciai, Paul E. Carlson, Shanmuga Sozhamannan, Alyxandria M. Schubert, Yi H. Yan

**Affiliations:** 1 MRIGlobal, 425 Dr. Martin Luther King Jr. Boulevard, Kansas City, MO 64110 USA; 2 Laboratory of Mucosal Pathogens and Cellular Immunology, Division of Bacterial, Parasitic and Allergenic Products, Office of Vaccines Research and Review, Biologics Evaluation and Research, U.S. Food and Drug Administration, Silver Spring, MD 20993 USA; 3 Division of Microbiology, Office of In Vitro Diagnostics, Office of Product Evaluation and Quality, Center for Devices and Radiological Health, U.S. Food and Drug Administration, Silver Spring, MD 20993 USA; 4 Defense Biological Product Assurance Office (DBPAO), Joint Program Executive Office for Chemical, Biological, Radiological and Nuclear Defense (JPEO-CBRND), Joint Project Lead, Enabling Biotechnologies JPL-EB, Frederick, MD 21702 USA; 5 Joint Research and Development, Inc., Stafford, VA 22556 USA

**Keywords:** Signature erosion, qPCR performance, *In silico* prediction, False negative result, Supervised learning, Diagnostic markers, Biological techniques, Biotechnology

## Abstract

Molecular assays are critical tools for the diagnosis of infectious diseases. These assays have been extremely valuable during the COVID pandemic, used to guide both patient management and infection control strategies. Sustained transmission and unhindered proliferation of the virus during the pandemic resulted in many variants with unique mutations. Some of these mutations could lead to signature erosion, where tests developed using the genetic sequence of an earlier version of the pathogen may produce false negative results when used to detect novel variants. In this study, we assessed the performance changes of 15 molecular assay designs when challenged with a variety of mutations that fall within the targeted region. Using data generated from this study, we trained and assessed the performance of seven different machine learning models to predict whether a specific set of mutations will result in significant change in the performance for a specific test design. The best performing model demonstrated acceptable performance with sensitivity of 82% and specificity of 87% when assessed using tenfold cross validation. Our findings highlighted the potential of using machine learning models to predict the impact of emerging mutations on the performance of specific molecular test designs.

## Introduction

Polymerase chain reaction (PCR)based molecular detection tests are widely used for the diagnosis of many infectious diseases^1,2^. These tests detect the presence of unique portions of pathogen genomes through targeted PCR amplification. Results for these PCR tests are used in both patient care and public health policy making. The accuracy of these tests largely depends on the primer and probe design of the tests, where mismatches to the primer and probe design have the potential for causing false negative results^3–6^. This is particularly true for molecular assays designed for the detection of viral targets with high mutation rates. For example, false negative results due to mismatches in the primer and probe regions of PCR-based molecular detection tests have been reported for both SARS-CoV-2^7–11^ and influenza diagnostic tests^12,13^.

With the rapid advance in genomic surveillance of infectious diseases^14–16^, public health officials and test manufacturers now have the capability to predict the emergence of specific variants and the associated mutations that fall within the regions targeted by PCR diagnostic tests^16^. However, not all mismatches in the primer and probe region of molecular diagnostic tests will cause false negative results. Previous studies have shown that the impact of mismatches on test performance is test design specific and affected by factors including template design, cycling conditions and buffer composition^17,18^. The ability to accurately predict the impact of mismatches on PCR test performance is needed to effectively leverage the available genomic surveillance data to identify PCR tests at risk of performance degradation due to emerging mutations.

Several different studies in the past have been conducted to characterize the impact of different types of mismatches on PCR performance^3–6,19–21^ through both *In silico* or experimental approaches. Previous *In silico* assessment of the impact of mismatches on molecular SARS-CoV-2 diagnostic tests have focused on alignment-based assessment^19–21^. Additional factors such as cycling conditions, buffer composition and mutation types can also impact PCR amplification efficiency^3–6^. Other experimental studies have been limited to the assessment of the impact of mismatches on the primer region of PCR assays and are often designed to evaluate the impact of individual substitutions on PCR assay performance. As observed during the COVID pandemic, mismatches to molecular diagnostic tests are not limited to single substitutions and could occur on both primers and/or the probe of molecular diagnostic tests. Numerous assessments of the impact of mismatches on SARS-CoV-2 test performance have been conducted^9,22,23^. However, because these studies are often carried out with different test designs, it is very difficult to leverage the results of previous studies for the training of generalizable predictive models to assess the impact of emerging mutations on test performance. Predicting the impact of emerging mutations on diagnostic tests is a challenging task due to the diversity of potential mismatches that could emerge over time, and the heterogeneity of existing mutation impact assessment data.

In this study, we aim to evaluate the potential of training a machine learning model to predict how specific mutations will impact the performance of PCR assays. A single PCR protocol was used to generate the training data for our model. We designed a mutation panel that consisted of 228 total SARS-CoV-2 PCR templates, based on publicly available primer and probe sequences of 15 assays designed to amplify specific regions of the SARS-CoV-2 genome. These 228 templates were designed to represent diverse types of mismatches that were observed during the COVID pandemic. Each mutation panel template, and the corresponding 15 wild type templates (without any mismatch), were amplified in triplicate, with the matching primer and probes at four different concentrations. The resulting cycles threshold (C*t)* values of mutation panel templates were compared to the C*t* values observed for the corresponding wild type template to quantify the impact of the specific mutations on PCR assay performance. These experiments produced a large quantitative dataset that was used to train several machine learning models to predict the impact of specific mutations on assay performance. Validation and analysis of the best performing model further identified features of mutations that have the most impact on assay performance and highlighted the limitations and potential of our model training approach.

## Methods

### Assay selection and wild-type templates

We tracked the performance of 43 SARS-CoV-2 qPCR assays throughout the pandemic periodically using an *In silico* analyses tool called PSET (PCR Signature Erosion Tool) against SARS-CoV-2 sequences from the Global Initiative on Sharing All Influenza Data (GISAID) database^19^. This tool used percent identity between the query sequence (assay signature sequences comprising primer, probe, and amplicon sequence) and the subject sequences from GISAID to identify emerging mutations with potential to cause false negative results for diagnostic assays.

Of the 43 assays tracked using PSET, 15 were identified as overlapping variant mismatches with high potential to cause decreased performance according to the PSET algorithm. We used these assays for this study because their designs covered a variety of gene targets, differed in their primer/probe sequences, and captures sequence design considerations for designing qPCR assays. We further selected a total of 228 unique mutation sets that were previously observed in the GISAID database for these 15 assays. The mutation sets were selected to represent a variety mutation types and features. These unique mutations were evaluated in our study using synthetic RNA or DNA templates. The assay design and their genomic locations are indicated in Table 1.

**Table 1:** Assay targets under investigation and their alignment position in the SARS-CoV-2 reference genome sequence (NC 045512.2).

Assay design	Forward primer (5′–3′)	Reverse primer (5′–3′)	Probe (5′–3′)	SARS-CoV-2 gene (alignment position)	# of mutated templates	References
C3 ORF3a	GTTACGACTATTGTATACCTTACAATAGTGTA	CACAGTCTTTTACTCCAGATTCCCATTTTTCA	AGGACTTGTTGTGCCATCACCTGAAG	ORF3a (25,830–26,013)	1	Unpublished (DNASoftware, Inc.)
C4 ORF8	CGTGTCCTATTCACTTCTATTCTAAA	ACTGTATAATTACCGATATCGATGTACTGA	TGGATGAGGCTGGTTCTAAATCACCC	ORF8 (27,980–28,155)	18	Unpublished (DNASoftware, Inc.)
CHAN S	CCTACTAAATTAAATGATCTCTGCTTTACT	CAAGCTATAACGCAGCCTGTA	CGCTCCAGGGCAAACTGGAAAG	S (22,692–22,889)	1	^24^
China N	GGGGAACTTCTCCTGCTAGAAT	CAGACATTTTGCTCTCAAGCTG	TTGCTGCTGCTTGACAGATT	N (28,861–28,999)	55	^25^
France IP2	ATGAGCTTAGTCCTGTTG	CTCCCTTTGTTGTGTTGT	AGATGTCTTGTGCTGCCGGTA	ORF1ab (12,670–12,817)	1	^26^
HKU ORF1b	TGGGGYTTTACRGGTAACCT	AACRCGCTTAACAAAGCACTC	TAGTTGTGATGCWATCATGACTAG	ORF1ab (18,758–18,929)	1	^27^
Young ORF1ab	TCATTGTTAATGCCTATATTAACC	CACTTAATGTAAGGCTTTGTTAAG	AACTGCAGAGTCACATGTTGACA	ORF1ab (14,135–14,263)	1	^28^
Young S	TATACATGTCTCTGGGACCA	ATCCAGCCTCTTATTATGTTAGAC	CTAAGAGGTTTGATAACCCTGTCCTACC	S (21,743–21,896)	10	^28^
NCOV N GENE	CACATTGGCACCCGCAATC	GAGGAACGAGAAGAGGCTTG	ACTTCCTCAAGGAACAACATTGCCA	N (28,686–28,853)	1	^29^
Noblis 40	GCCGCTGTTGATGCACTATG	TGTCGTCTCAGGCAATGCAT	ACGTGCTCGTGTAGAGTGTTTTGAT	ORF1ab (17,150–17,357)	1	^30^
Japan N2	AAATTTTGGGGACCAGGAAC	TGGCAGCTGTGTAGGTCAAC	ATGTCGCGCATTGGCATGGA	N (29,125–29,282)	16	^31^
BVP 501Y	CGGTAGCACACCTTGTAATG	ACTACTACTCTGTATGGTTGGTAA	CCCACTTATGGTG	S (22,986–23,096)	10	^32^
CDC N1	GACCCCAAAATCAGCGAAAT	TCTGGTTACTGCCAGTTGAATCTG	ACCCCGCATTACGTTTGGTGGACC	N (28,267–28,377)	51	^33^
CDC N2	TTACAAACATTGGCCGCAAA	GCGCGACATTCCGAAGAA	ACAATTTGCCCCCAGCGCTTCAG	N (29,144–29,249)	57	^33^
Yale 69/70 del	TCAACTCAGGACTTGTTCTTACCT	TGGTAGGACAGGGTTATCAAAC	TTCCATGCTATACATGTCTCTGGGA	S (21,700–21,839)	4	^34^

### Mutated templates

**Fig. 1:**
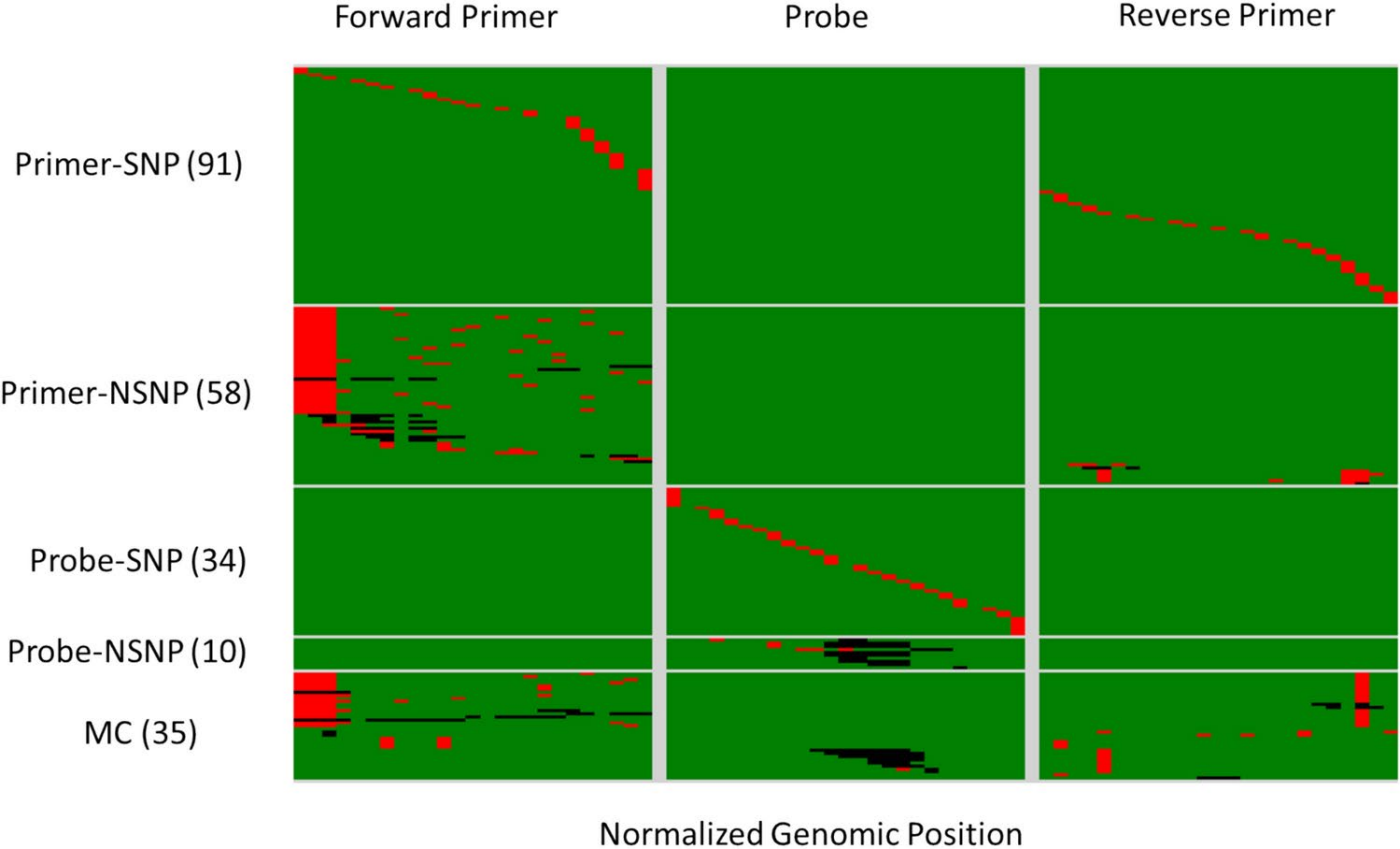
Visual summary of mutated templates tested. Each row represents a single mutated template. The templates are grouped by location and mutation template type shown in Table 2. Green tiles represents perfect alignment to primer/probe sequence, red tiles indicate a substitution at that position and black tiles indicate a deletion.

For this study, we quantified the impact of 228 mutation sets that fall within the primer and/or probe binding region of the 15 assay targets (Table 1) on qPCR performance using DNA templates. The use of DNA template is appropriate for this study because the goal of the study is to specifically characterize the impact of mismatches within the target template on qPCR amplification and mismatches are unlikely to hinder reverse transcription. The mutations introduced were picked from observed SARS-CoV-2 mutations in the GISAID database and designed to cover a wide range of observed mutation types. Mutation template types tested in the study is described in Table 2. The relative position of mismatches within each mutated template tested in this study is shown in Fig. 1. Detailed descriptions of each of the targets, with their primer, probe, and template sequences, can be found in Supplementary File 1.

**Table 2:** Description of types of mutation templates tested.

Mutation template type	Description	# of templates
Primer-SNP	Single Nucleotide Polymorphism (SNP) within one of the primer regions, no mutation on any other component	91
Primer-NSNP	Non-SNP mutations (i.e., deletion, multi-nucleotide substitution) within one of the primer region, no mutation on any other component	58
Probe-SNP	SNP within the probe region, no mutation on any other component	34
Probe-NSNP	Non-SNP mutations (i.e., deletion, multi-nucleotide substitution) within the probe region, no mutation on any other component	10
MC	Multicomponent Mutations on more than one PCR component	35

### PCR design and experiments

Mutated templates and wild-type (positive control) templates were ordered as synthetic DNA oligos (gBlock fragments) from IDT and included 20 base pairs of flanking sequence on each end of the template. The templates were tested at four initial concentrations (50, 500, 5000, and 50,000 copies per reaction) with 3 replicate reactions per level, alongside no template controls (NTCs) consisting of molecular grade water and positive controls (Wild Type Templates). A universal set of reagents and thermocycling parameters was used for testing all assays (Table 1), which included TaqPath 1-Step RT-qPCR Master Mix, CG (Thermo Fisher Cat. No. A15299). Primers and probes (IDT; PrimeTime™ 5′ 6-FAM™/ZEN™/3′ IB^®^FQ) were included in the reaction at final concentrations of 900 nM and 250 nM, respectively. These concentrations were selected because these are the highest concentrations recommended by the manufacturer for this master mix and are therefore likely the most permissive of mismatches. The final reaction volume was 20 µL: 15 µL of master mix with 5 µL of template added. The thermocycling protocol used is shown in Table 3. This protocol corresponds to the manufacturer’s recommended protocol for this master mix, with two modifications: (1) the number of cycles was increased from 40 to 50 to allow generation of C*t* values for templates with suboptimal amplification efficiency due to mismatches or deletions, and (2) the annealing/extension temperature was reduced from 60 to 55 °C to be more permissive of mismatches and be reflective of the annealing/extension temperature recommended for many of the published assays evaluated.

**Table 3:** PCR cycling conditions.

Step	Temperature (°C)	Time	Cycles
Reverse transcription	50	15 min	1
Denaturation	95	2 min	1
Annealing/extension	95	3/5* s	45*/50
	55	30 s	

*CDC N1 and N2 templates were amplified with slightly altered melting time and total cycle number. For the purpose of this study, we have analyzed all the qPCR results together.

PCR was performed on a Bio-Rad CFX96 real-time PCR instrument, and post-PCR analyses were performed using a universal threshold to assess mutant template performance when compared with the wild-type template. In addition to qualitative results (detection or no detection), quantitative performance metrics evaluated include amplification efficiency, linear regression coefficient (R^2^), y-intercept, and average C*t* values at each template concentration tested.

Synthetic DNA oligos for CDC N1 and N2 template regions (wild-type and mutant templates) were synthesized by Genscript USA and were tested using pre-mixed N1 and N2 primer/probe sets from IDT 2019-nCoV EUA diagnostic panel kits (1.5 µl per 20 µl reaction). PCR amplification of the CDC N1 and N2 templates were performed in triplicates with minor changes to the melting time and total PCR cycle (Table 3).

### Classification model training and validation

For our data analysis and model training, each mutated template is described with 13 feature variables shown in Table 4. These variables were chosen based on a literature review to include well-known features of primer/probe mismatches that are likely to impact qPCR performance^3–6,17,21^. Additionally, because this study included mutated templates that have mismatches on both primers, we also included additional features to describe these multi-primer mismatches (i.e. second primer features). Feature representation of each template and associated C*t* values are included in Supplementary File 1.

**Table 4:** Feature descriptions.

Features represented within mutated templates		
Feature description	Feature abbreviation	Notes
Percent of substitutions in the first primer	% MM P1	If a template only has mismatches in a single primer, that primer is labeled as first primer. If both primers contain mismatches, then primers will be randomly labeled as first primer and the other second primer
Percent of substitutions in the second primer	% MM P2	
Percent of substitutions in the probe	% MM Probe	
Percent of deletion in the first primer	# Del P1	
Percent of deletion in the second primer	# Del P2	
Percent of deletion in the probe	# Del Probe	
Nucleotide distance from the 3′ end of the first primer to the nearest substitution/deletion	Nearest 3′ P1	
Nucleotide distance from the 3′ end of the second primer to the nearest substitution/deletion	Nearest 3′ P2	
Number of deletion and substitution within 5 nucleotide distance to the 3′ of the first primer	# MM/Del 3′ P1	
Number of deletion and substitution within 5 nucleotide distance to the 3′ of the second primer	# MM/Del 3′ P2	
Difference in annealing temperature of the first primer and protocol annealing temperature (55 °C)	Temp Diff P1	Annealing temperatures were calculated using the Tm_GC function of the biopython package^35^
Difference in annealing temperature of the second primer and protocol annealing temperature (55 °C)	Temp Diff P2	
Difference in annealing temperature of probe and protocol annealing temperature (55 °C)	Temp Diff Probe	

For each template concentration, the difference in C*t* value (ΔC*t* value) of a mutated template in comparison to the wild-type template is calculated using the following formula:

$$\Delta {\text{C}}t\,{\text{value }} = \, \left( {{\text{Average}}\,{\text{ C}}t_{{{\text{Mutated}}}} } \right) \, {-} \, \left( {{\text{Average }}\,{\text{C}}t_{{\text{Wild - Type}}} } \right)$$

For each template concentration, if a mutated template failed to produce a positive PCR result or had ΔC*t* greater than a chosen threshold (ΔC*t* > 1/3/5) the PCR result of the mutated template at that initial concentration was labeled as “Not Significantly Changed,” otherwise, it was labelled as “Significantly Changed.” If a mutated template had more than one “Significantly Changed” qPCR result among the 4 initial concentrations, that template was labeled as “Significantly Changed.” An example of how the PCR result of each mutated template is labeled is shown in Table 5 below.

**Table 5:** Scheme for making actionable calls of PCR results.

	50 Copies/reaction	500 Copies/reaction	5000 Copies/reaction	50,000 Copies/reaction	Template label
Mutated Template 1	Significantly changed	Not significantly changed	Not significantly changed	Not significantly changed	Not significantly changed
Mutated Template 2	Significantly changed	Significantly changed	Significantly changed	Not significantly changed	Significantly changed
Mutated Template 3	Significantly changed	Significantly changed	Not significantly changed	Not significantly changed	Significantly changed

We applied seven different machine algorithms (Fig. 3) to build seven different models to predict whether a template would be classified as “Significantly Changed” or not using the 16 features described in Table 4 as input. Data from all 228 mutation templates were used for model training and validation. Leave-one-out cross-validation (LOOCV), tenfold cross validation (10FCV) and leave-one-assay-out cross validation (LOAOCV) were used to estimate model performance. LOOCV is a configuration of k-fold cross validation where “k” is set to the number of samples in the dataset. LOAOCV is a configuration of cross-validation where during each cross validation, mutation templates from one of the 15 assay designs were left out of model training and used for validation. Model performance was assessed using area under the receiver operating characteristic curve (AUROC), sensitivity, and specificity. All statistical analyses were performed with Scientific Python 3.6 libraries (Scikit-Learn 1.4.0^36^).

## Results

### Ct value difference observed in mutated templates

**Fig. 2:**
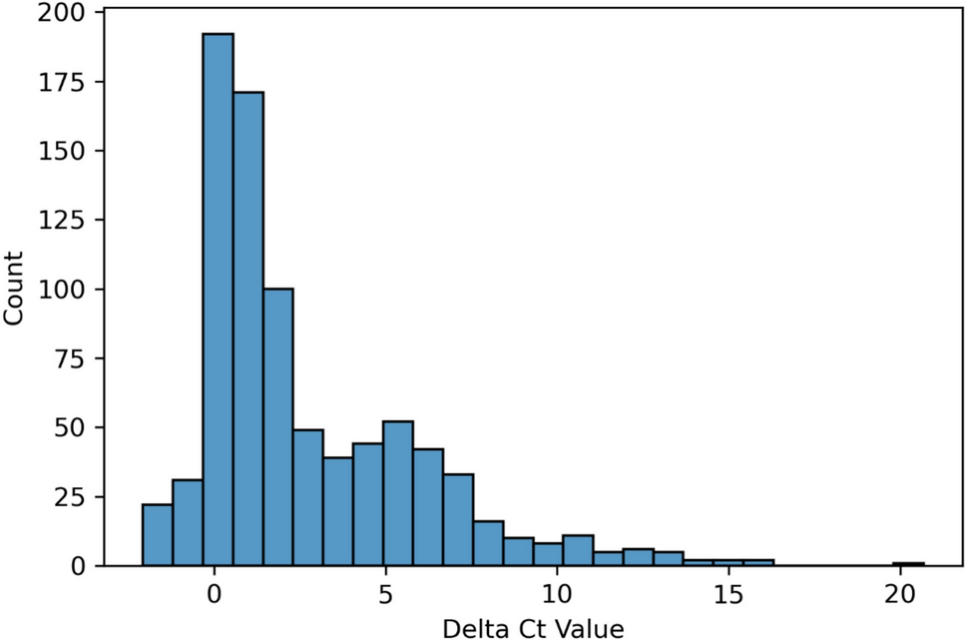
Histogram of ΔC*t* values.

ΔC*t* values for each mutated template were calculated for each template concentration. A positive ΔC*t* value indicates a decrease in analytical sensitivity of a qPCR assay for the detection of the mutated template in comparison to the wild-type (i.e., it took more cycles of amplification for the mutated template to reach the fluorescence intensity threshold for detection in comparison to the wild-type template). We calculated a total of (228 mutated templates) x (4 template concentrations) = 912 ΔC*t* values. Of these, 34 templates did not produce a detection result in at least 1 concentration and in total accounted for 60 ΔC*t* values labeled “Not Detected.” In this study, the majority (96.9%) of ΔC*t* values were greater than -1. We have observed an average ΔC*t* value of 2.70 in our study with 285 ΔC*t* values greater than 3. The distribution of ΔC*t* values observed in this study is shown in Fig. 2.

To assess the correlation between mutated template types and ΔC*t* values, we calculated the average ΔC*t* values for each of the mutated template types as described in Table 2. The results are shown in Table 6.

**Table 6:** Average ΔC*t* values for each type of mutation template.

Mutation template type	Avg C*t* value change, (10% Quantile to 90% Quantile), # of ΔC*t* values
Primer-SNP	1.994 (− 0.300 to 6.482), 299
Primer-NSNP	4.423 (0.585 to 8.471), 210
Probe-SNP	0.73 (− 0.350 to 1.89), 192
Probe-NSNP	2.558 (0.570 to 4.241), 20
MC	4.653 (0.165 to 9.908), 122

We observed that non-SNP templates have significantly higher ΔC*t* values than SNP templates for both primers and probe (*p* < 10^–10^). Additionally, SNP and non-SNP mutations on primers have significantly higher ΔC*t* values than those same mutation types on the probe (*p* < 10^–6^). However, the 10%–90% quantile of ΔC*t* values for each mutation type is generally very wide often ranging from negative ΔC*t* values to ΔC*t* values greater than 5, indicating that template mutation type is likely a poor predictor for ΔCt value.

We have also calculated the Spearman’s correlation (rho) between each of the 16 representative features of the mutated templates and their associated ΔC*t* values. These results are shown in Table 7.

**Table 7:** Spearman’s correlation of ΔC*t* values and mutation template features.

Feature	Spearman’s Rho	*P* value
#MM/DEL 3′ P1	0.327	6.09E−24
% Del P1	0.302	1.73E−20
% MM P1	0.266	4.64E−16
% MM P2	0.254	8.96E−15
% Del Probe	0.196	2.69E−09
Temp Diff P2	0.160	1.25E−06
#MM/DEL 3′ P2	0.064	5.41E−02
% Del P2	− 0.070	3.49E−02
Nearest 3′ P2	− 0.215	6.30E−11
% MM Probe	− 0.274	4.32E−17
Temp Diff P1	− 0.323	1.70E−23
Temp Diff Probe	− 0.400	3.63E−36
Nearest 3′ P1	− 0.432	2.03E−42

None of the features showed a strong correlation (< 0.5 or > 0.5) with ΔC*t* values. Based on these results, we hypothesized that mutation type and individual mutation features offer limited predictive power for ΔC*t* values, despite being associated with ΔC*t*, as demonstrated by the significant *P*-values (Table 7).

### Significantly changed templates

As we observed in the previous ΔC*t* values analysis, only a minority of the mutations tested caused complete non-detection of the target (34 out of 228 templates, or ~ 15%). The majority of the mutations tested caused an increase of the C*t* value instead. A positive ΔC*t* value is likely to result in an increase in the limit of detection of the assay and its impact on clinical performance of the assay would depend on both the viral load distribution in the infected population and the magnitude of ΔC*t*. Therefore, a single ΔCt value to determine significantly changed qPCR performance is likely not representative of real-world testing scenarios. To understand how mutation type impacts the change of qPCR performance broadly, we determined the number of significantly changed mutation templates based on four pre-specified thresholds. The results are shown in Table 8.

**Table 8:** Number of significantly changed templates according to 4 different thresholds among 5 different mutation template type.

	Primer-SNP	Primer-non-SNP	Probe-SNP	Probe-non-SNP	MC
No detection (ND)	0/77	7/58	0/48	5/10	6/35
ΔC*t* > 1 or ND	41/77	50/58	18/48	9/10	30/35
ΔC*t* > 3 or ND	20/77	38/58	1/48	8/10	23/35
ΔC*t* > 5 or ND	17/77	28/58	0/48	5/10	18/35

Each mutation template was labeled as “Significantly Changed” or “Not Significantly Changed” for each of the four ΔC*t* thresholds: “ND”, “ΔC*t* > 1 or ND,” “ΔC*t* > 3 or ND,” and “ΔC*t* > 5 or ND.” These definitions are used in the subsequent training and validation of seven machine learning models.

### Model comparison

**Fig. 3:**
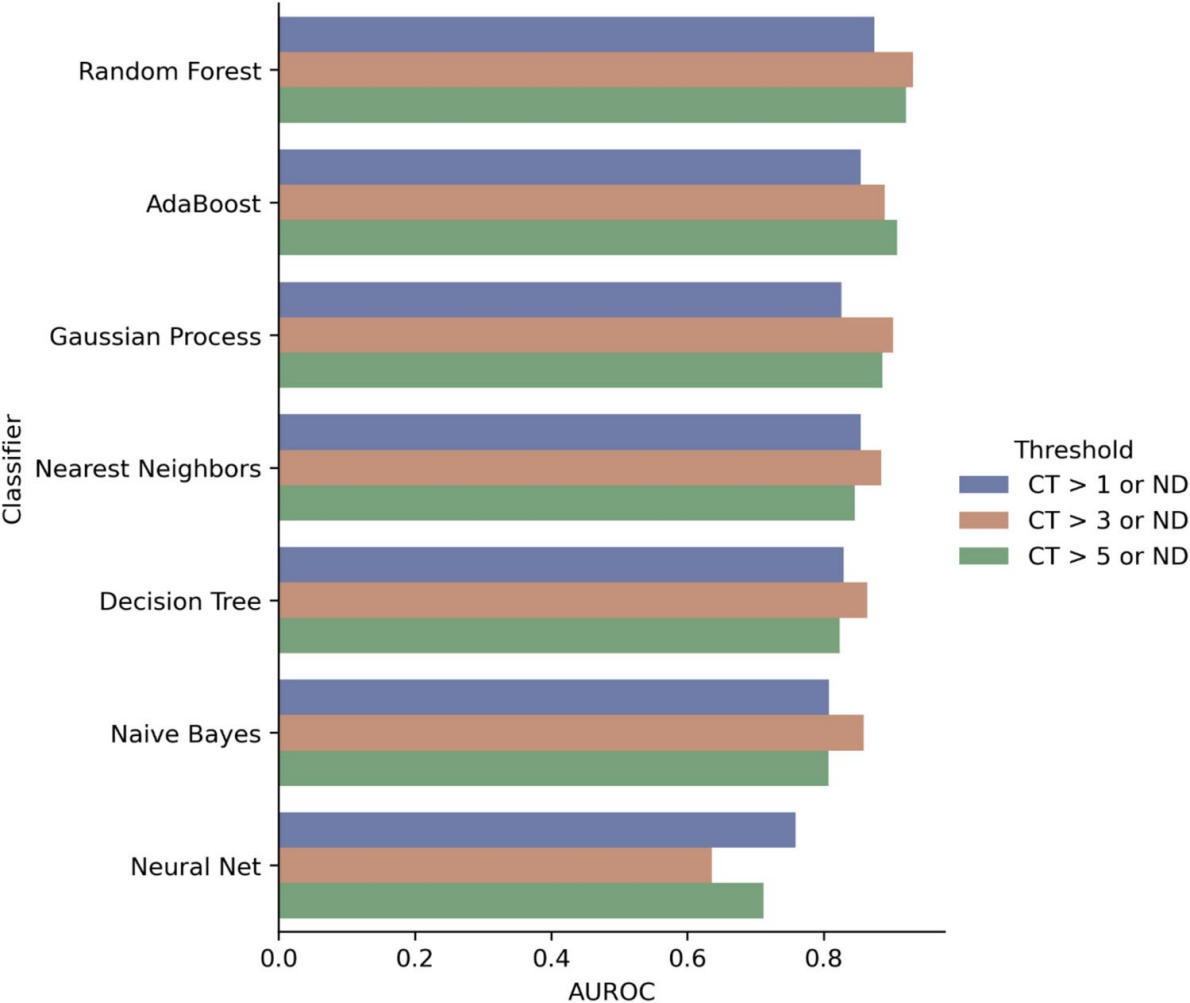
10FCV AUROC of different classifiers with different ΔC*t* thresholds (Numerical results used for Fig. 3 can be found in Supplementary File 2).

We utilized tenfold cross validation (10FCV) area under the receiver operand curve (AUROC) of each model to compare the performance of the seven supervised learning models trained to predict whether specific mutations will lead to significant change in C*t* value during qPCR amplification. AUROC was calculated for each model and each of the three thresholds for determining significant change: “ΔC*t* > 1 or ND,” “ΔC*t* > 3 or ND,” and “ΔC*t* > 5 or ND.” The “ND” threshold was not used in model training due to the small number of templates that were labeled as “Significantly Changed” under the ND threshold (18). The results are shown in Fig. 3.

The best performing classifier was the Random Forest classifier, which had an average AUROC of 0.91 across all three thresholds. Five out of seven models had the highest AUROC with the “ΔCt > 3 or ND” threshold. Based on this observation, we used the results of the “ΔCt > 3 or ND” threshold for additional model robustness analysis.

### Model robustness

**Fig. 4:**
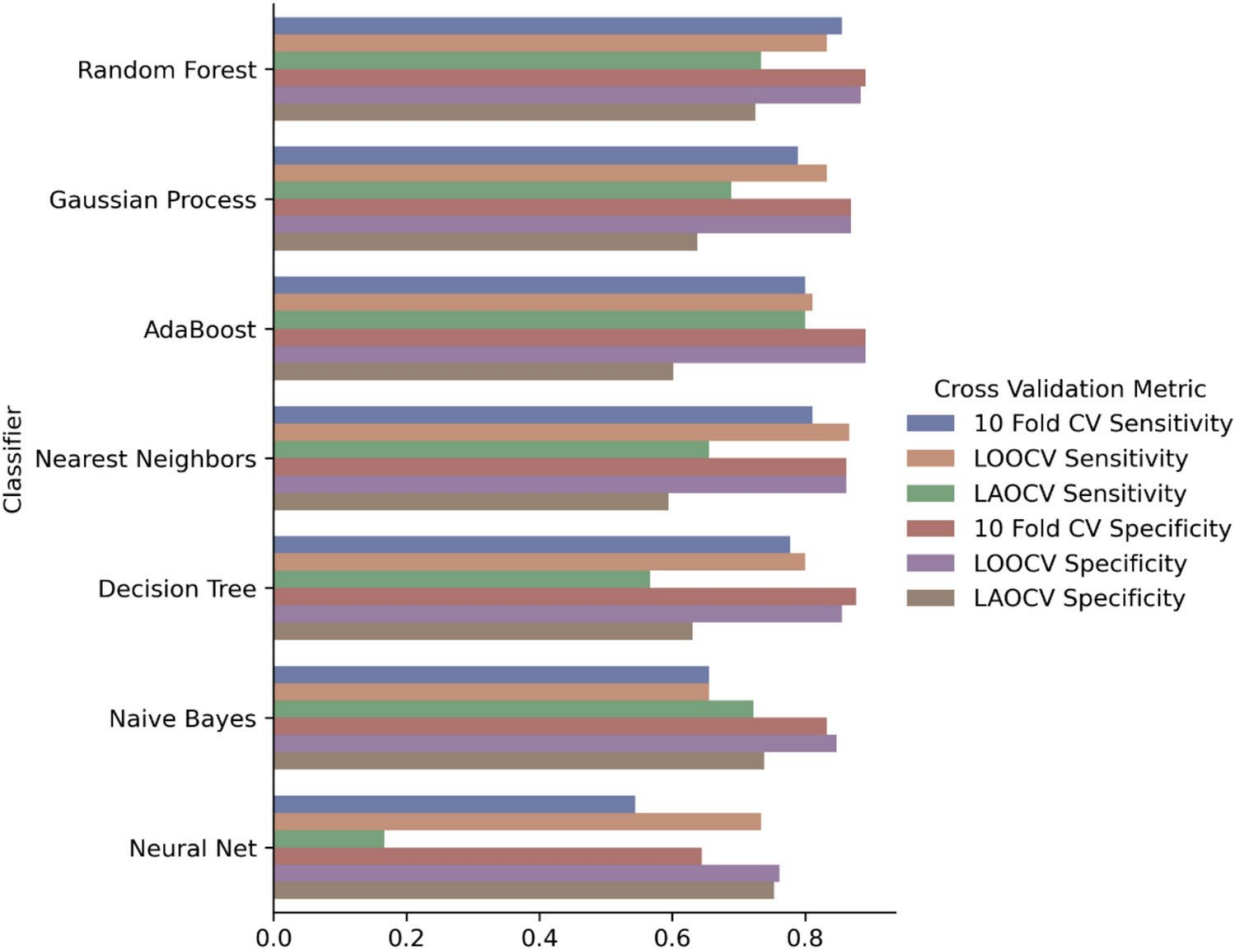
Sensitivity and specificity of models with different cross validation approaches using the “ΔC*t* > 3 or ND” threshold. (Numerical results used for Fig. 4can be found in Supplementary File 2).

The robustness of our trained models was assessed based on its generalizability to unseen mutations within templates used to train the model and to unseen templates. Classifier performance estimated with the tenfold cross validation and Leave One Out Cross Validation (LOOCV) method assesses the generalizability of the models to unseen mutations when the base primer/probe template have been used for model training. Classifier performance estimated with Leave One Assay Out Cross Validation (LOAOCV) assesses the generalizability of the models to primer/probe mutation sequences never trained on by the model (Fig. 4). The robustness analysis results for the “ΔC*t *> 3 or ND” classification are shown in the following figure:

The model performance estimated with 10FCV and LOOCV approaches are very similar across all the classifiers evaluated. The Random Forest classifier (RF) demonstrated the highest 10FCV AUROC of 0.93 with 10FCV sensitivity of 85.5% and specificity of 89.1%.

In comparison to the performance estimated with 10FCV and LOOCV, LOAOCV produced significantly worse performance for all models, resulting in a large drop in specificity for six out of the seven models. The only model that did not have significant drop in LOAOCV specificity (i.e., Neural Net) also displayed a large drop in LOAOCV sensitivity (~ 60%). RF has shown LOAOCV sensitivity of 73.3% compared to the 85.5% 10FCV sensitivity. RF also has LOAOCV specificity of 72.4%, which is significantly worse than the 10FCV specificity of 89.1%.

### Assay level performance

To assess whether the variation of the number of templates available for each of the 15 assays included in this study could impact the assay specific performance of the trained models, we have calculated the assay specific sensitivity and specificity for the random forest model trained using the “ΔC*t* > 3 or ND” threshold. The results are shown below Table 9.

**Table 9:** Assay specific sensitivity and specificity.

Assay	Total # of templates	# of significantly changed templates	LOOCV sensitivity	LOOCV specificity	LOAOCV sensitivity	LOAOCV specificity
CDC-N2	57	17	0.76	0.90	0.06	1.00
China N	55	39	0.87	0.56	0.97	0.00
CDC-N1	51	4	0.75	0.98	1.00	0.79
C4 ORF8	18	5	0.80	1.00	1.00	0.46
Japan_N2	14	0	NA	1.00	NA	1.00
BVP 501Y	10	6	1.00	0.50	1.00	0.00
Young-S	10	6	0.67	0.50	0.50	0.75
Yale 69/70 del	4	4	1.00	NA	1.00	NA
Japan_N	2	2	1.00	NA	0.00	NA
C3 ORF3a	1	1	1.00	NA	1.00	NA
CHAN S	1	1	0.00	NA	0.00	NA
France_IP2	1	1	1.00	NA	1.00	NA
HKU ORF1b	1	1	0.00	NA	0.00	NA
NCOV-N-GENE	1	1	1.00	NA	1.00	NA
Noblis.40	1	1	1.00	NA	1.00	NA
Young-ORF1ab	1	1	1.00	NA	1.00	NA
All	228	90	0.83	0.88	0.73	0.72

We did not observe significant correlation between the Total # of Templates for each assay and their LOOCV/LOAOCV sensitivity and specificity (*Spearman’s correlation, p-value* > *0.05*). The CDC-N2 assay has shown the largest difference in assay specific model performance when comparing the result of the LOOCV and LOAOCV.

### Feature importance

**Fig. 5:**
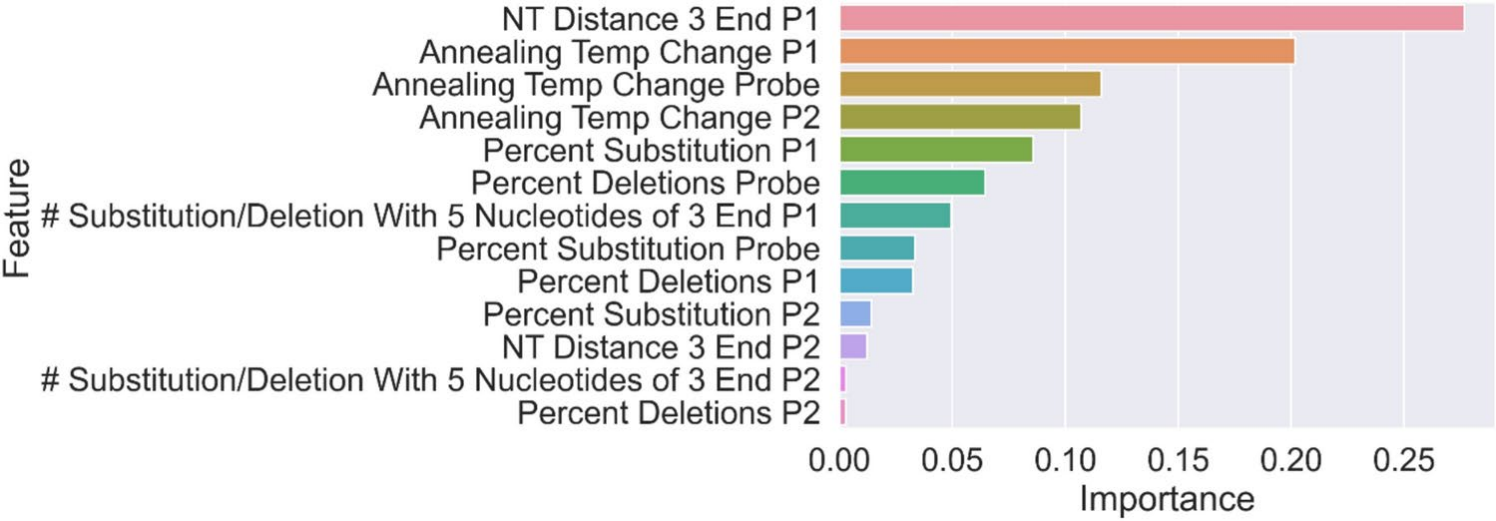
Feature importance of the RF Model for threshold “ΔC*t* > 3 or ND”.

To better understand how the random forest model interpreted the features to make predictions, a feature importance analysis was conducted for the random forest model trained using the “ΔC*t *> 3 or ND” threshold. The feature importance value was calculated with the “feature_importances_” function of the “Scikit-Learn” Python package^36^. Feature importance measures the contribution of each identified feature (i.e. characteristic) for the classifier to accurately function. The results are shown in Fig. 5.

The majority of P2 features are shown to have low feature importance for the random forest model trained using the “ΔCt > 3 or ND” threshold. Because the P2 features are unique to mismatches that impact both the forward and reverse primers and are not well represented in our training data (11.4%, 26/228), we do not believe that the low feature importance shown here is indicative of the actual impact of P2 mismatches on qPCR performance.

## Discussion

Multiple studies have shown that mismatches near the 3′ end of primers and mismatches that would cause significant annealing temperature changes could be extremely detrimental to PCR detection^3,4^. Our current study not only confirmed these previous findings but also improved the assessment of mismatch impact on PCR performance through the use of machine learning models. Our study demonstrated that individual aspects of mismatches, such as number of mismatches near the 3′ end of the primer and estimated annealing temperature change due to mismatches, are significantly correlated with ΔC*t*. However, the correlation is not strong enough for accurate prediction of ΔC*t* with any individual features. The 10FCV performance of our trained model demonstrated the potential of accurately predicting the impact of mismatch using multiple features of the mismatch. With the best performing model demonstrating a 10FCV sensitivity of 85.5% and specificity of 89.1% for predicting whether a set of mismatches would result in ΔC*t* > 3 or amplification failure.

In this study we were able to build several machine learning models for predicting the impact of unseen mutations on primer/probe designs that have being used as a part the training data. The best performing models has estimated AUROC > 0.9 and 10FCV sensitivity and specificity both greater than 85% for predicting whether a set of mismatches would result in ΔCt > 3 or amplification failure. The feature importance analysis of the random forest model shows that the distance of the mismatches to the 3′ end of primers and changes in annealing temperature due to the mismatches in both primers and probe are the top three most important features for the model. This finding aligns with previous research^3–6^ and lends confidence that the model is operating in a manner that is consistent with current mechanistic understanding of qPCR reactions.

However, as shown in the LOAOCV study, the performance of the models decreased significantly when cross validated with mismatches that fall on primer/probe designs not used for training of the models. The top performing models demonstrate LOAOCV sensitivity of 73.3% and specificity of 72.4%, a large decrease from the 10FCV sensitivity of 83.3% and specificity of 89.1% for predicting whether a set of mismatches would result in ΔC*t* > 3 or amplification failure. Although the number of templates for each assay used to train the models are highly variable, we did not observe significant correlations between the available training data for each assay and the assay specific performance (Table 9). Therefore, we believe that the significant difference in model performance between the two cross validation methods demonstrates that the model training methodology and feature representation utilized in this study are unlikely to be generalizable to primer/probe designs not seen in the training data. We hypothesize that this is likely because the impact of mismatches on primer and/or probe binding is both nucleotide-specific and local sequence-specific, i.e., mismatches to primer/probe sequences are not captured by the features used to represent mismatches in the current study. For example, in our study, we have observed that the C29197G mutation in the CDC N2 Probe would cause a significant shift in C*t* value (ΔC*t* > 3) but not the C29197T^11^ mutation which occurs in the same position (ΔC*t* < 1). Additional features that capture these aspects of mismatches have the potential to increase the generalizability of our predictive method.

In this study, we also tested two mutations that have been reported to cause detection failures in diagnostic assays. The C29200T and C29197T mutation observed in the CDC N2 Probe^11^ and the G29234A mutation observed in the Japanese CDC N2 probe^10^. In our study, none of the mutations caused detection failure and showed a ΔC*t* < 2 for all the samples tested. We hypothesize that this difference is likely due to the differences in PCR parameters e.g., cycling protocol, master mix components, and primer/probe concentrations) used in our study compared to study that reported detection failure. For the C29200T mutation, the detection failure has only been reported for the Cepheid Xpert Xpress Instrument, and the Japanese N failure was reported in a test that used different instruments and reagents compared to our study. This discrepancy suggests that the impact of mismatches on qPCR amplification and detection is likely instrument and PCR protocol specific. Models trained using data from a specific PCR protocol may not be generalizable to other PCR instruments or protocols.

Based on the results of our study, we conclude that the feature representation and model training algorithm tested in this study are likely suitable for training models to predict the impact of novel mismatches on primer/probe designs that were included in model training. Similar approach would likely be applicable to all PCR diagnostic tests regardless of the targeted organism or specimen type. However, as demonstrated by the large drop in model performance when using the LOAOCV, novel feature representation of mismatches that are representative of specific nucleotide differences and PCR parameters would likely be needed to train a generalizable model to predict the impact of novel mismatches on primer and probe design not used in model training. Despite the limitations of our modelling approach, it could still provide public health benefit by predicting emerging mutations that are most likely to cause signature erosion in widely used tests, especially for viruses with a high mutation rate such as Influenza or SARS-CoV-2.

## Supplementary Information

Supplementary Information.

Supplementary Information.

## Data Availability

All data generated or analysed during this study are included in this published article.
